# P-432. Understanding Weight Changes in HIV Patients on Integrase Strand Transfer Inhibitor-based Antiretroviral Therapy: Insights from Southern India

**DOI:** 10.1093/ofid/ofae631.632

**Published:** 2025-01-29

**Authors:** Ashin Siby, Sanju Biju, Shon Paulson

**Affiliations:** Azeezia Medical College Hospital, Paika, Kerala, India; St. Joseph's College of Pharmacy, Thrissur, Kerala, India; St. Joseph's College of Pharmacy, Thrissur, Kerala, India

## Abstract

**Background:**

As HIV infection continues to pose a significant public health challenge, especially in regions like Southern India, effective antiretroviral therapy (ART) remains pivotal for improving patient outcomes. Integrase Strand Transfer Inhibitors (INSTIs) have emerged as cornerstone agents in ART regimens, yet their specific impact on weight changes in HIV-infected individuals, particularly in Southern India, warrants comprehensive investigation due to limited existing data.

**Methods:**

This retrospective study analyzed medical records of HIV-infected patients receiving care at healthcare facilities in Southern India. Patients who initiated INSTI-based antiretroviral therapy between April1, 2022, and December 31, 2023, were included in the analysis. Data on demographic characteristics, baseline weight, and weight measurements at follow-up visits were collected from electronic medical records. Statistical analysis, including descriptive statistics and paired t-tests, was conducted to assess changes in weight over time following initiation of INSTI-based therapy.

**Results:**

The study included 84 patients diagnosed with HIV, predominantly HIV-1, with a male representation of 61% (n=51) and a median age of 36.3 years (range: 20–65 years). Common opportunistic infections observed were pulmonary tuberculosis, oral candidiasis, and herpes zoster. The primary antiretroviral therapy regimens were TDF+3TC+DTG or TDF+FTC+DTG. Following initiation of INSTI-based therapy, 69% of patients experienced weight gain, averaging 3.81 kg at 3 months, with 18.1% gaining over 4 kg. Median BMI increased from 23.82 kg/m² to 25.9 kg/m² at 8.5 months post-ART initiation. Among ART-naïve patients, 76% experienced weight gain, with 18.1% gaining 8.36 kg at 9 months. Gender and age did not show significant associations with weight gain. However, weight gain exhibited a positive correlation with time after INSTI initiation (r = +1), indicating an expected increase in weight per 0.1 months (F = 4.62, p = 0.032).

**Conclusion:**

In conclusion, our findings underscore the significant weight gain associated with INSTI-based antiretroviral therapy among HIV-infected patients in Southern India, emphasizing the importance of monitoring and managing metabolic changes in clinical practice.

**Disclosures:**

**All Authors**: No reported disclosures

